# Knockdown of SLC39A7 suppresses cell proliferation, migration and invasion in cervical cancer

**DOI:** 10.17179/excli2017-690

**Published:** 2017-10-24

**Authors:** Yongqing Wei, Jie Dong, Fuli Li, Zhuqing Wei, Yuling Tian

**Affiliations:** 1 Department of Gynecology and Obstetrics, Jinan Central Hospital Affiliated to Shandong University, Jinan 250012, China; 2 Department of Stomatology, Chengyang People's Hospital, Qingdao 266109, China

**Keywords:** cervical cancer, SLC39A7, cell proliferation, apoptosis, migration, invasion

## Abstract

Cervical cancer is the fourth leading cause of malignancy related mortality in women worldwide. SLC39A7 (ZIP7) is a zinc transporter that plays a key role in intestinal epithelial self-renewal. However, whether or not SLC39A7 is involved in human cervical cancer remains unclear. In this study, we investigated the effects of SLC39A7 in cervical cancer* in vitro* and elucidate related underlying mechanisms. Using Oncomine data analysis, we first found SLC39A7 is commonly upregulated in cervical cancer tissues in comparison with corresponding normal controls. The *in vitro *experiments indicated that silencing of SLC39A7 expression resulted in decreased cell proliferation, increased cell apoptosis, and attenuated migratory and invasive ability using CCK-8, colony formation, flow cytometry, transwell assays, respectively in cervical cancer cell lines, HeLa and ME-180 cells. In molecular levels, Western blot further demonstrated that silencing of SLC39A7 significantly upregulated the expression of Bax and E-cadherin, downregulated the expression of Bcl-2 and MMP-2 in both HeLa and ME-180 cells. These findings provide evidence that SLC39A7 plays a positive role in the progression of cervical cancer and its knockdown might be as a potential therapeutic target for cervical cancer treatment.

## Introduction

Cervical cancer has been regarded as a lethal disease attacking women around the world, accounting for an estimated 528,000 new cases and 266,000 deaths in 2012 (Ferlay et al. 2015[8]; Schiffman et al. 2007[31]). Several risk factors have been linked to cervical cancer, including human papillomavirus (HPV), smoking, and dysfunction of the immune system, especially HPV 16 and 18 which lead to approximately 60 % to 70 % of cervical cancers (Khan et al. 2005[16]; Kjellberg et al. 2000[17]). The cure rate of cervical cancer is associated with invasive stage of tumor progression, most patients can be cured at early stage, whereas the late stage is generally incurable (Stevanovic et al., 2015[35]). Previous study showed that about 67 % percent of the patients are diagnosed at late stage, chemoradiotherapy is currently considered to be the major nursing measure to cervical cancer, however, the prognosis remains poor and largely ineffective (Taylor and Gercel-Taylor, 2008[36]). Thus, an increased understanding of the molecular mechanism of cervical cancer progression and metastasis is essential for improving clinical treatment strategies. 

Zinc is an indispensable trace element necessary for mitotic cell division, enzyme synthesis, transcription regulation, DNA and RNA synthesis, playing key roles in multiple biochemical processes (Prasad, 1983[27]; Yan et al., 2012[46]). More than 3000 proteins required zinc as a cofactor, which account for 10 % of the genome, including zinc-finger family member (Andreini et al., 2006[1]; Taylor et al., 2012[37]). The pathophysiology of multiple diseases are associate with abnormal zinc accommodation, such as diabetes mellitus (Kazi et al., 2008[15]), neurodegenerative disorders (Cuajungco and Lees, 1997[6]), and chronic inflammation and immunity (Prasad, 2009[28]). It is worth noting that zinc accumulation is closely related to several types of cancer (Taylor et al., 2008[40]). In comparison with benign breast tissue, human breast cancer samples showing zinc hyper-accumulation (Taylor et al., 2008[40]), while malignant cells in prostate cancer virtually lost ability to accumulate zinc, which is manifested at a relatively low levels of zinc (Costello et al., 2004[5]). The rodent mammary tumorigenesis induced by **N**-methyl-**N**-nitrosourea had strikingly increased 19-fold levels of zinc relative to the normal counterparts (Nandi et al., 1995[23]).

The homeostasis of cytosolic zinc is tightly controlled by zinc exporters (Slc30a/ ZnT) and importers (Slc39a/Zip) engaged in zinc accumulation, exportation, and intracellular compartmentalization (Eide, 2004[7]; Palmiter and Huang, 2004[26]). Within the Slc39a/Zip family, the SLC39A7 (ZIP7) protein is predominately resided in endoplasmic reticulum (ER) membrane, the Golgi, or both two of them (Huang et al., 2005[12]; Taylor et al., 2004[38]). It has been shown to release of zinc from the endoplasmic reticulum and mediate the cell signaling activation that contributes to cell proliferation (Taylor et al., 2007[39]). Taylor et al. (2012[37]) showed that phosphorylation of the conserved residues in the SLC39A7 results in tyrosine kinases activation, AKT phosphorylation, and ERK1/2 signaling pathway. Studies identifying upregulation of SLC39A7 increased intracellular zinc pools with the result of activation of growth factor, leading to promotion of tamoxifen-resistant MCF-7 cells viability (Taylor et al., 2008[40]). SLC39A7 expression is remarkably upregulated in gastric tumor model, also play an essential role for intestinal homeostatic self-renewal (Itadani et al., 2009[13]; Ohashi et al., 2016[25]). It can be activated upon extracellular stimulation via phosphorylation by protein kinase casein CK2 which involved in biological processes such as proliferation, migration, apoptosis and mitosis (St-Denis and Litchfield, 2009[34]; Taylor et al., 2012[37]). We hypothesized that SLC39A7 might be implicated in the growth and aggressive behavior of cervical cancer cells. 

Here, we demonstrated that knockdown of SLC39A7 leaded to decreased proliferation, migration and invasion, and induced apoptosis in cervical cancer cells. In addition, deficient in SLC39A7 expression exhibited decreased expression of Bcl-2 and MMP-2, and increased expression of Bax and E-cadherin. Our results firstly revealed that SLC39A7 may play a vital function in cervical cancer carcinogenesis by regulating apoptotic and epithelial to mesenchymal transition (EMT) progresses.

## Materials and Methods

### Oncomine datasets analysis

SCL39A7 gene expression was analyzed using microarray gene expression datasets derived from the Oncomine database (http://www.oncomine.org). To determine the differential expression of SCL39A7 between cervical cancer and their normal counterparts, the results were filtered by defining Cancer Type was as cervical cancer, Data Type was mRNA, and Analysis Type was Cancer versus Normal Analysis. Comparison statistical analysis was conducted using Oncomine algorithms.

### Cell lines and culture

Human cervical cancer cell lines (HeLa, SiHa, CaSki, ME-180), human embryonic kidney 293 T (HEK393T) and a normal human cervical epithelial cell line (H8) were obtained from the American Type Culture Collection (Manassas, VA, USA). They were cultured in DMEM with 10 % fetal bovine serum (FBS, Gibco Laboratories, USA) and maintained at 37 °C in an atmosphere of 5 % CO_2_.

### Plasmid construction and cell transfection

Three short hairpin RNA (shRNA) sequences (shRNA-SCL39A7#1, #2 and #3) targeting the coding region of SCL39A7 gene and a negative control (NC) sequence were purchased from OriGene Technologies, Inc. (Rockville, MD). These oligonucleotides were annealed and cloned into pLVX-shRNA vector sites between *EcoRI* and *BamHI* restriction sites. Lentivirus particles were generated by co-transfecting recombined vector (sh-SLC39A7#1, sh-SLC39A7#2 and sh-SLC39A7#3) and packing vector into HEK293T cells using Lipofectamine 2000 (Invitrogen, Carlsbad, CA, USA). For cell transfection, HeLa and ME-180 cells were cultured in 6-well plates and transfected with lentivirus supernatant at a multiplicity of infection (MOI) of 10. After 48 h transfection, quantitative real-time PCR (qRT-PCR) and Western blotting was performed to determine if the knockdown was effective.

### RNA isolation and qPCR

Total RNA was extracted using the Trizol reagent (Invitrogen, Carlsbad, CA). The complementary DNA (cDNA) synthesis was performed using SuperScript II RT 200 U/Ml (Invitrogen) from 2 μg of total RNA. Real-time PCR was performed on the Applied Biosystems 7500 Real-Time PCR System (ABI, USA) with SYBR Premix Ex Taq (Takara, JPN) in final 20 µL reaction volume, including 2 µL cDNA, 10 µL SYBR Green Master Mix, 0.5 µL each of the forward and reverse primers (10 pmol), and 7 µL nuclease-free water. The relative expression level of SLC39A7 mRNA was normalized to that of the endogenous control (GAPDH). Data was analyzed using the 2^-ΔΔCt ^method. PCR for each sample was performed in triplicates. 

### Western blotting

Total protein was extracted from cells using RIPA lysis buffer and quantified using protein quantification reagents from Bio-Rad. Equal amount of proteins (20-40 μg) were electrophoresed on 10 % sodium dodecyl sulfate-polyacrylamide gel (SDS-PAGE) and transferred to a PVDF membrane by electroblotting. After blocking with 5 % non-fat dry milk for 1 h at room temperature, the membranes were probed with the corresponding primary antibodies against SLC39A7, Bax, Bcl-2, E-cadherin, MMP-2 and GAPDH overnight at 4 °C. Subsequently, the membrane was incubated with appropriated horseradish peroxidase-conjugated secondary antibodies (Santa Cruz Biotechnology, Santa Cruz, CA) for 2 h at room temperature. The blots were visualized using super ECL detection reagent (Applygen, Beijing, China).

### Cell Counting Kit-8 (CCK8) assay

The cell viability was assessed using the CCK-8 assay. In brief, approximately 2 × 10^3^ cells were reseeded into 96-well plates and incubated for 8 h. Then, 10 μl CCK-8 solution (Dojindo, Japan) was added to each well and incubated at 37 °C for 4 h. Finally, the optical density (OD) value in each well was detected at a wavelength of 450 nm at indicated time point (24, 48, 72 and 96 h post-seeding). Experiments were performed in triplicate.

### Colony formation assay

Transfected cells were seeded into six-well plates at a density of 600 cells per well. After cultured for 14 days in complete growth media, naturally formed colonies were fixed with cold methanol and stained with 0.4 % crystal violet for 30 min. The colonies (50 cells per colony) were manually counted under a microscope. Experiments were performed in triplicate.

### Cell apoptosis analysis

Cell apoptosis was analyzed by flow cytometry with Annexin V-APC/7-AAD Apoptosis Detection Kit (Key GEN BioTECH). Briefly, transfected cells were reseeded in 6 cm dishes at 8 × 10^4^ cells per dish. Then cells were collected and washed twice with PBS, and subjected to Annexin V-APC/7-AAD double staining according to the manufacturer's protocol. The percentage of apoptotic cells were determined by FAC Scan flow cytometry (Becton-Dickinson, CA, USA). Experiments were performed in triplicate.

### Cell migration and invasion assays

After 48 h transfection, approximately 2 × 10^5^ HeLa and ME-180 cells were in 150 µl per well were plated in the upper chamber (8.0 μm, Costar, USA) with a porous membrane without Matrigel solution in FBS-free medium. Then, medium containing 10 % FBS as a chemoattractant was added to the lower chamber. After 24 h of incubation at 37 °C, cells migrating to the lower surface of the chamber were fixed with 4 % paraformaldehyde and stained with 0.1 % crystal violet for 2 h. Total five random visual fields were selected and the average was calculated. The cell invasion assay was simultaneously performed with the above steps, except that the porous membrane was pre-coated with Matrigel solution (BD, Franklin Lake, USA). Experiments were independently performed at least three times.

### Statistical analysis

All statistical analyses were performed using GraphPad Prism 5.0 and the quantitative data were shown as mean ± standard deviation (SD). Student's t-test was performed to compare differences between two groups. P value of less than 0.05 was considered as statistical differences.

## Results

### SCL39A7 expression is upregulated in cervical cancer tissues

**Figure 1:**
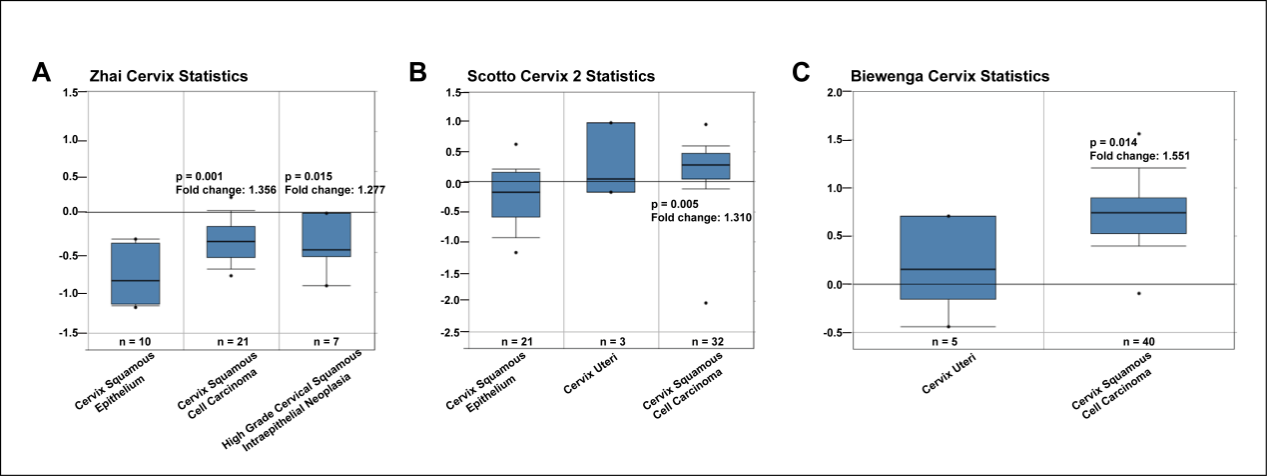
SCL39A7 is upregulated in cervical cancer tissues. The mRNA expression of SCL39A7 was analyzed in Oncomine datasets including (A) Zhai Cervix, (B) Scotto Cervix 2 and (C) Biewenga Cervix.

To investigate the association between SCL39A7 and cervical cancer progression, the differential mRNA expression of SCL39A7 was determined between cervical cancer tissues and normal cervix tissues by analysis of the Oncomine microarray gene expression datasets. The results showed that the expression of SCL39A7 was significantly increased in cervix squamous cell carcinoma tissues compared with the corresponding normal cervix tissues in three datasets, including Zhai Cervix (Figure 1A(Fig. 1), *p* = 0.001, *p* = 0.015) (Zhai et al., 2007[49]), Scotto Cervix 2 (Figure 1B(Fig. 1), *p* = 0.005) (Scotto et al., 2008[32]) and Biewenga Cervix (Figure 1C(Fig. 1), *p* = 0.014) (Biewenga et al., 2008[3]). The data highlighted there might be a close relation between overexpressed SCL39A7 and cervical cancer progression.

### Downregulation of SCL39A7 by shRNA transfection in cervical cancer cells

**Figure 2:**
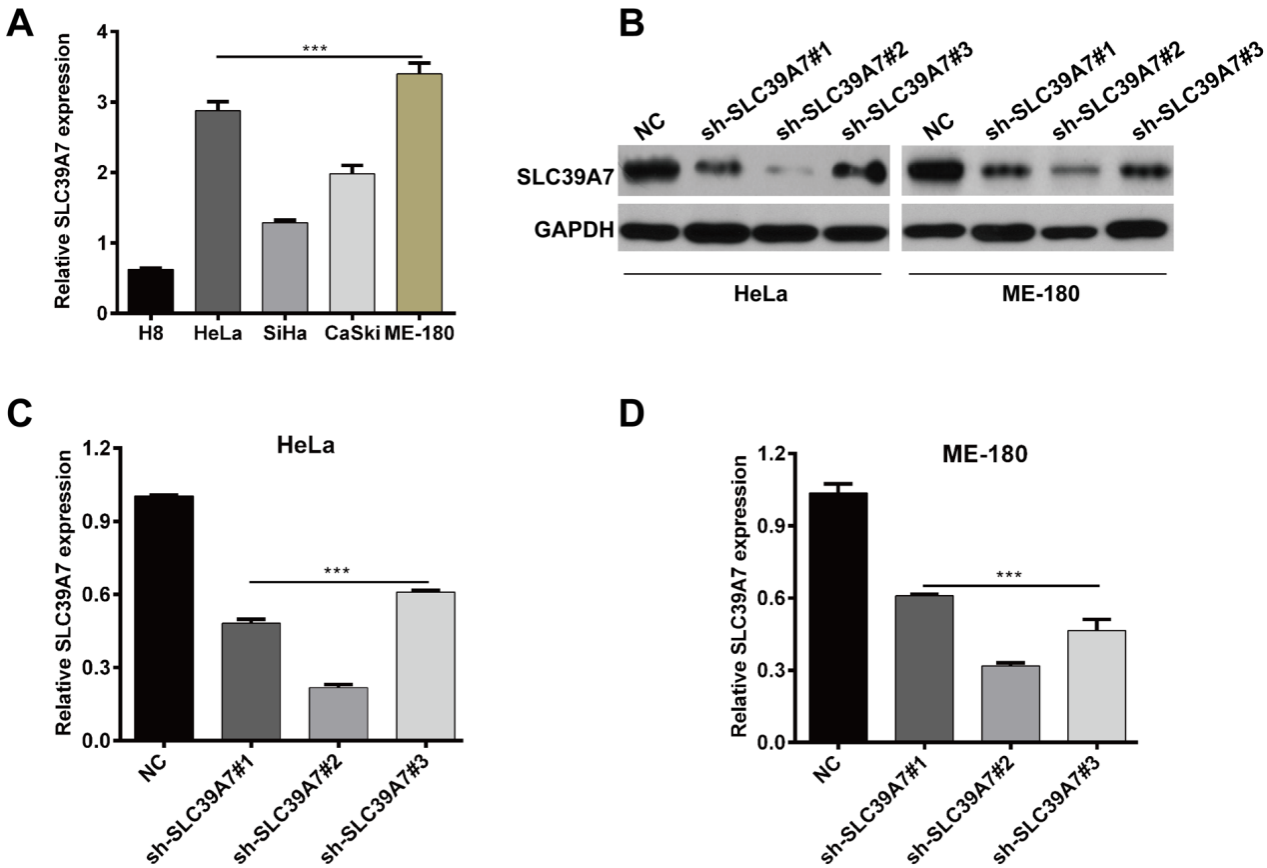
Silencing of SCL39A7 expression in cervical cancer cells by sh-SCL39A7 transfection. (A) The expression of SCL39A7 mRNA was determined in several cervical cancer cell lines (HeLa, SiHa, CaSki, ME-180) and a normal cervical epithelial cell line H8 by qRT-PCR analysis. (B) Western blot analysis of SCL39A7 protein levels in HeLa and ME-180 cells following sh-SCL39A7#2 infection. The qRT-PCR analysis of SCL39A7 mRNA levels in HeLa (C) and ME-180 (D) following sh-SCL39A7#2 infection; Values were expressed as mean ± standard deviation (SD). ***p < 0.001 compared to negative control (NC)

Subsequently, we aimed to explore the function of SLC39A7 in cervical cancer *in vitro*. As shown in Figure 2A(Fig. 2), the expression of SLC39A7 was found to be higher expressed in cervical cancer cell lines (HeLa, SiHa, CaSki, ME-180) than in normal cervical epithelial cell line H8 (*p* < 0.001). To performed loss-of-function assays, three shRNA was designed to knockdown SLC39A7 expression in HeLa and ME-180 which have the highest of SLC39A7 expression. As shown in Figure 2B(Fig. 2), sh-SLC39A7#2 is the most efficient one to downregulate SLC39A7 expression in both HeLa and ME-180 cell lines by Western blot analysis. Consistently, qRT-PCR also confirmed sh-SLC39A7#2 is the efficient sequence in HeLa (Figure 2C(Fig. 2), *p* < 0.001) and ME-180 (Figure 2D(Fig. 2), *p* < 0.001) cell lines, as used for the following experiments.

### Downregulation of SCL39A7 suppressed cell proliferation in cervical cancer

**Figure 3:**
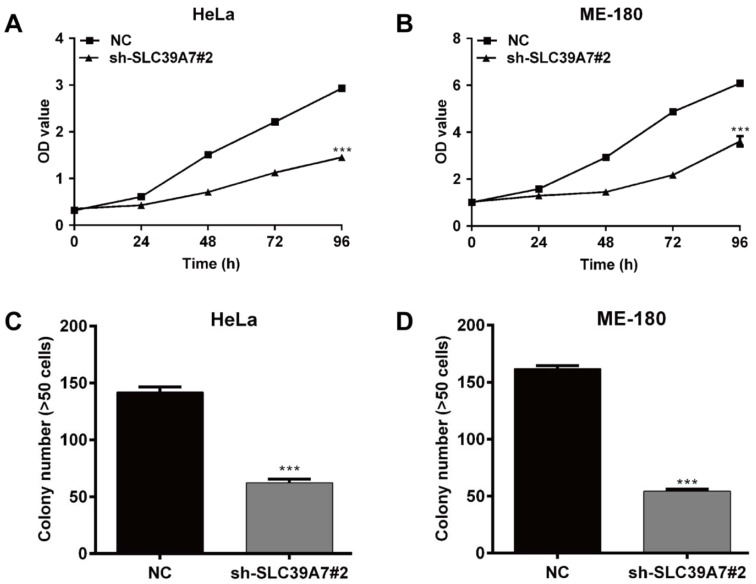
Silencing of SCL39A7 inhibited cell proliferation in cervical cancer cells. CCK-8 assay was used to determine cell viability in HeLa (A) and ME-180 (B) following sh-SCL39A7#2 infection. Colony formation assay was performed to evaluate cell proliferation ability in HeLa (C) and ME-180 (D) following sh-SCL39A7#2 infection. Values were expressed as mean ± standard deviation (SD). ***p < 0.001 compared to negative control (NC)

To investigate whether SLC39A7 plays a role in cell proliferation in cervical cancer, the CCK-8 assay was used to evaluate cell viability in HeLa and ME-180 cells following sh-SLC39A7#2 transfection. As shown in Figure 3A(Fig. 3) and 3B(Fig. 3), the cell viability was significantly lower after consecutive 5 days in SLC39A7 knockdown (sh-SLC39A7#2) cells compared with non-target transfected cells (NC) in HeLa and ME-180 cells (*p* < 0.001).

In addition, colony formation assay was performed to gain insight into the effect of SLC39A7 knockdown on cell proliferation. As shown in Figure 3C(Fig. 3) and D(Fig. 3), SLC39A7 knockdown obviously reduced the number of colonies formed in HeLa and ME-180 cells (*p* < 0.001).

### Downregulation of SCL39A7 promoted cell apoptosis in cervical cancer

**Figure 4:**
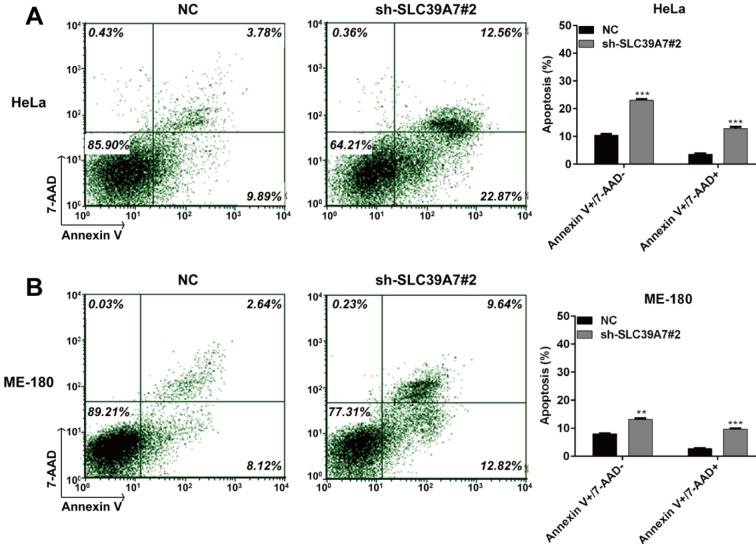
Silencing of SCL39A7 induced apoptosis in cervical cancer cells. Cell apoptosis was analyzed by Annexin V/7-AAD staining and flow cytometry in HeLa (A) and ME-180 (B) cells following transfection with lentivirus containing sh-SCL39A7#2 or NC. Values were expressed as mean ± standard deviation (SD). ***p < 0.001 compared to negative control (NC)

Next, we explored the underling mechanism of impaired cell proliferation by SLC39A7 knockdown by analyzing cell apoptosis. As shown in Figure 4A(Fig. 4), both early apoptotic (Annexin V+/7-AAD-) and late apoptotic (Annexin V+/7-AAD+) cells in sh-SLC39A7#2 transfected cells increased significantly compared with those in NC-transfected cells in HeLa cells (*p* < 0.001). Similar results were also observed in ME-180 cells (Figure 4B(Fig. 4), *p* < 0.001).

### Downregulation of SCL39A7 inhibited cell migration and invasion in cervical cancer

**Figure 5:**
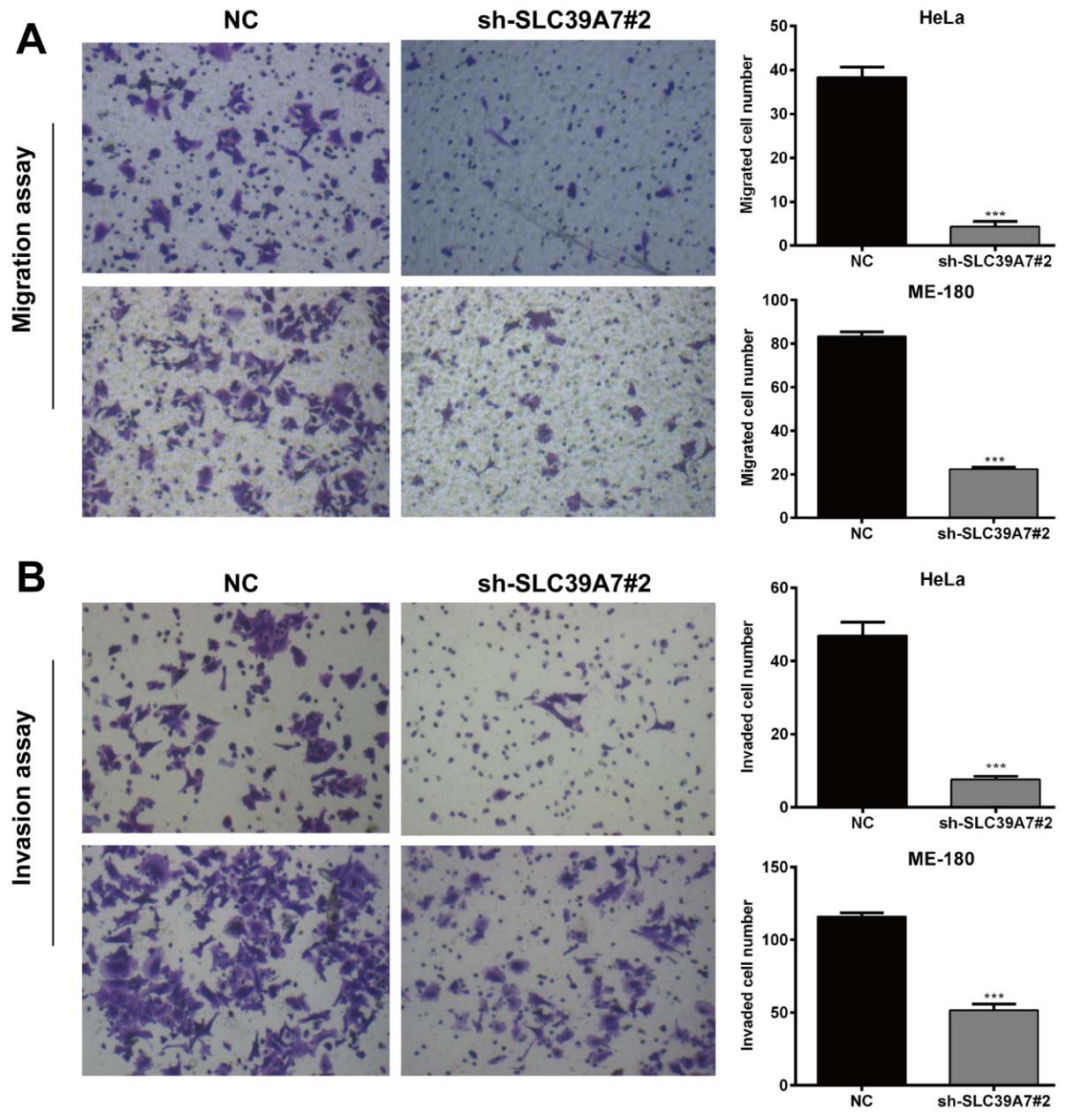
Silencing of SCL39A7 suppressed cell migration and invasion in cervical cancer cells. (A) Effect of SCL39A7 knockdown on cell migration of HeLa and ME-180 after sh-SCL39A7#2 transfection. (B) Effect of SCL39A7 knockdown on cell invasion of HeLa and ME-180 after sh-SCL39A7#2 transfection. Values were expressed as mean ± standard deviation (SD). ***p < 0.001 compared to negative control (NC)

Furthermore, the effects of SCL39A7 on the ability of cell migration and invasion were determined in cervical cancer cells by Transwell assay. As shown in Figure 5A(Fig. 5), migration assay showed that the number of migratory cells in sh-SLC39A7#2 treated groups was dramatically decreased compared with those in the NC group in HeLa and ME-180 cells (*p* < 0.001). In the invasion assay, the invaded cells were also decreased significantly after SCL39A7 knockdown in HeLa and ME-180 cells (Figure 5B(Fig. 5), *p* < 0.001).

### Downregulation of SCL39A7 regulated apoptotic and EMT markers in cervical cancer

**Figure 6:**
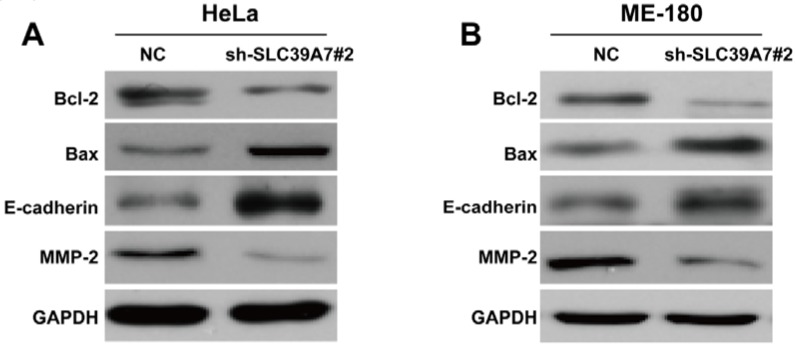
Effect of SCL39A7-silencing on its downstream molecular associated with cell apoptosis and migration regulation in HeLa (A) and ME-180 (B), as confirmed by Western blot analysis. GAPDH was used as internal control.

Subsequently, we detected the expression alterations of some apoptosis and EMT markers. As shown in Figure 6A(Fig. 6) and 6B(Fig. 6), the sh-SLC39A7#2 transfection obviously decreased anti-apoptotic Bcl-2 expression and increased pro-apoptotic Bax expression in HeLa and ME-180 cells. Besides, EMT marker, E-cadherin was significantly upregulated, while MMP-2, associated with invasion was downregulated in HeLa and ME-180 cells following sh-SLC39A7#2 transfection.

## Discussion

Cervical cancer, a highly fatal magligancy, has now become the second most common cancer for women in the world (Moss and Blaser, 2005[21]). SLC39A7 is a member of the LIV-1 subfamily of zinc transporters, and role of aberrant SLC39A7 expressions are relative to cell growth and death (Myers et al., 2013[22]). Previous studies prompted us to explore the impact of SLC39A7 on the biological behavior of cervical cancers. In the current study, we have characterized the tumor promoter activity of SLC39A7 in cervical cancer cells. SLC39A7-deficient mediated aggressive growth inhibition and apoptosis induction correlates with the alteration in expression of Bcl-2, Bax, MMP-2 and E-cadherin. 

Apoptosis, a physiological programmed cell death that is necessary for maintaining normal tissues homeostasis, the realization that the capability of cell escape apoptosis is a typical “characteristic of cancer” (Hanahan and Weinberg, 2000[10]). The apoptosis pathway can be regulated by pro-and anti-apoptotic molecules. Among them, anti-apoptotic proteins Bcl-2, Bcl-XL, and Mcl-1 of the Bcl-2 family, and the pro-apoptotic proteins Bid, Bax, and Bad attract widespread attention (Korsmeyer et al., 1999[18]; Willis et al., 2005[43]). Following an apoptotic stimulus, Bax and Bad could mediate induction of mitochondrial outer permeability transition, triggering the release of cytochrome c from mitochondria and other cell-death-associated molecules, while Bcl-2 opposing Bax and Bad to preserve outer membrane integrity (Yip and Reed, 2008[47]). When cells facing apoptosis stimuli, the survival or apoptosis are determined by the ratio of Bcl-2/Bax, which considered as a cell-autonomous rheostat, that non-apoptotic cells usually have a higher ratio than apoptotic cells (Korsmeyer et al., 1995[19]). In this study, knockdown of SLC39A7 inhibited Bcl-2 expression but increased Bax expression, leading to lowered ratio of Bcl-2/Bax, which caused acceleration of apoptosis. Previous studies demonstrated that zinc protects cell against oxidative stress, and zinc fluxes are closely related to apoptosis (Truong-Tran et al., 2000[41]). Zinc accumulation has been proven to increase the Bcl-2/Bax ratio, thereby contributing to decrease the sensitivity of the cells to apoptosis (Fukamachi et al., 1998[9]). Singla and Dhawan (2015[33]) showed a protection role of zinc for neurotoxicity induced by AL though stimulating anti-apoptotic activity including elevated the ratio of Bcl-2/Bax. SLC39A7 play important roles in preserving intracellular zinc homeostasis though modulation the reapportionment of zinc from intracellular zinc pool to the cytosol (Colvin et al., 2008[4]). In tamoxifen-resistant MCF-7 cells, depletion of SLC39A7 decreased the activation of epithelial growth factor receptor (EGFR), IGF-I receptor (IGF-1R) and c-Src signaling due to reduced cellular zinc levels (Taylor et al., 2008[40]). We supposed that the intracellular zinc pools were also decreased in SLC39A7-knockdown cervical cancer cells compared with control, resulting in downregulation of the Bcl-2/Bax ratio, hence allowing acceleration of apoptosis. 

In addition, SLC39A7-attenuation induced a significant increase in the levels of E-cadherin and decrease in the levels of MMP-2 in comparison to control. EMT is increasing recognized as a driver of carcinoma invasion, migration, and metastasis (Biddle and Mackenzie, 2012[2]). It is the involvement of several steps, including the loss of cell-cell junctions between epithelial cells and activation of cell migration (Yamada and Cukierman, 2007[45]). E-cadherin downregulation has been considered as an essential process for epithelial to mesenchymal transdifferentiation, it occurs during tumor cells invade into the adjacent tissues (Sanchez-Tillo et al., 2010[30]). E-cadherin binding with α, β, and γ-catenin and is directly involved in the building actin cytoskeleton (Sanchez-Tillo et al., 2010[30]). Defective in E-cadherin/catenin complex is majorly implicated in cancer development and progression (Van Aken et al., 2001[42]). The downregulation of E-cadherin is associated with tumor aggressiveness, carcinogenesis, migration, and poor clinical outcome (Nawrocki-Raby et al., 2003[24]). In a previous report, ZEB1 could promote EMT though depressing the expression of E-cadherin by interaction with the brahma related gene 1 (BRG1). Krüppel-like Factor 4 increases E-cadherin expression in mammary epithelial cells and suppress EMT to prevent breast cancer cells migration and invasion (Yori et al. 2010[48]). Interestingly, Hershfinkel et al. (2010[11]) have revealed that there is a close linkage between zinc transporter and E-cadherin, that hyper expression of ZIP6 (SLC39A6) and ZIP10 (SLC39A10) promotes cell detachment and migration, also attenuates E-cadherin levels which in turn accelerated metastasis. Jin et al. (2015[14]) showed that knockdown of ZIP5 (SLC39A5) in human esophageal cancer cells elevates E-cadherin expression, contributing to repression of proliferation, migration and invasion. We found that SLC39A7 may play a role in regulating E-cadherin, like other zinc transporters ZIP5, 6, and 10. In the present study, stable knockdown of SLC39A7 increased the expression of E-cadherin, indicating the suppression of EMT of cervical cancer cells, leading to inhibition of invasiveness, proliferation and colony-formation. 

High expression of MMPs is largely implicated in the ability of epithelial cells to obtain an invasiveness phenotype (Nawrocki-Raby et al., 2003[24]). Previous studies revealed that E-cadherin-transfected cells displayed a decrease of MMP-2 activity (Miyaki et al., 1995[20]; Rajavashisth et al., 1999[29]). Recent seminal articles have pointed out that MMP-2 expression may link to certain types of zinc transporters. Specific overexpression of the zinc transporter ZIP4 resulted in an increase in MMP-2 in hepatocellular carcinoma (Xu et al., 2014[44]). In our experiments, the protein expression of MMP-2 was dramatically reduced in cervical cancer cells with SLC39A7 knockdown, probably as a result of overexpression of E-cadherin, thus promoted the invasion and metastasis of these cells. We supposed that SLC39A7 may indirectly regulate MMP-2 by altering E-cadherin expression in cervical cancer. 

In conclusion, the zinc transporter SLC39A7, whose abundance is increased in cervical cancers, and knockdown of SLC39A7 mediate inhibition of cell proliferation, migration, invasion and induction of apoptosis though upregulation of Bax and E-cadherin and downregulation of Bcl-2 and MMP-2, supports a metastasis promoted role for SLC39A7 in cervical cancer. Deeper understanding the molecular events that responsible for SLC39A7 knockdown-induced apoptosis and growth inhibition, may supply a more rational strategy to anticancer exploration.

## Acknowledgement

None.

## Conflict of interest

We all declare that we have no conflict of interest.
